# A Review of Sperm Ultrastructural Characters in the Opecoelidae (Digenea) and Their Phylogenetic Implications, with New Data on *Peracreadium characis*, a Parasite of *Diplodus puntazzo* in Tunisia

**DOI:** 10.3390/ani13121953

**Published:** 2023-06-10

**Authors:** Hichem Kacem, Jordi Miquel

**Affiliations:** 1 Laboratoire de Biodiversité Marine et Environnement, Département des Sciences de la Vie, Faculté des Sciences de Sfax, Université de Sfax, BP 1171, Sfax 3000, Tunisia; hichem.kacem@fss.usf.tn; 2 Secció de Parasitologia, Departament de Biologia, Sanitat i Medi Ambient, Facultat de Farmàcia i Ciències de l’Alimentació, Universitat de Barcelona, Avgda. Joan XXIII, sn, 08028 Barcelona, Spain; 3 Institut de Recerca de la Biodiversitat (IRBio), Universitat de Barcelona, Avgda. Diagonal, 645, 08028 Barcelona, Spain

**Keywords:** *Peracreadium characis*, Opistholebetinae, Opecoelidae, Digenea, spermatozoon, ultrastructure

## Abstract

**Simple Summary:**

The mature spermatozoon of *Peracreadium characis*, an intestinal parasite of the sheephead bream, is described by means of transmission electron microscopy (TEM). It shares the common ultrastructural features described in the majority of digeneans (two 9+‘1’ axonemes, mitochondria, nucleus and parallel cortical microtubules). The absence of external ornamentation of the plasma membrane and spine-like bodies distinguishes the spermatozoon of *P. characis* from the spermatozoa of the remaining studied opecoelids.

**Abstract:**

The spermatozoon ultrastructure of *Peracreadium characis* (Stossich, 1886) (Digenea: Opecoelidae), an intestinal parasite of the sheephead bream *Diplodus puntazzo* (Walbaum, 1792) (Sparidae), is described by means of transmission electron microscopy (TEM). The mature spermatozoon possesses two axonemes of the 9+‘1’ trepaxonematan pattern, an anterior electron-dense material, two mitochondria, a nucleus and parallel cortical microtubules distributed in two bundles. The absence of external ornamentation of the plasma membrane and spine-like bodies are the noteworthy characters that distinguish the spermatozoon of *P. characis* from those of most opecoelids. In fact, only *Helicometra fasciata* lacks external ornamentation in the spermatozoon. A comparative study with the remaining opecoelids described so far reveals similarities in the ultrastructural organization of their sperm cells. In addition, the current data on sperm ultrastructure in species of the recognized opecoelid subfamilies are compared, namely the Hamacreadiinae, Helicometrinae, Opecoelinae, Opistholebetinae and Plagioporinae.

## 1. Introduction

The cosmopolitan digenean family Opecoelidae Ozaki, 1925 is a species-rich family with more than 90 genera and almost 900 species, parasitizing the digestive tract of marine and freshwater teleost fishes and also sporadically found in amphibians [1].

The phylogenetic relationships and the taxonomic structure of Opecoelidae trematodes has always been controversial and repeatedly subject to change. Cribb [2] included this family within the Allocreadioidea Looss, 1902 and emphasized that the status of the superfamily was not stable. Later, Curran et al. [3] incorporated the first molecular data for representatives of the Allocreadiidae Looss, 1902 into broader analyses of the Xiphidiata Olson, Cribb, Tkach, Bray & Littlewood, 2003. They synonymized the Allocreadioidea with the Gorgoderoidea Looss, 1901 and proposed that the families previously included under the Allocreadioidea (i.e., Opecoelidae + Opistholebetidae Fukui, 1929; Brachycladiidae Odhner, 1905 and Acanthocolpidae Lühe, 1906) should be grouped under the superfamily Brachycladioidea Odhner, 1905. Subsequently, Littlewood et al. [4] removed the Opecoelidae from the superfamily Brachycladioidea, recognizing opecoelids as a superfamily on their own, the Opecoeloidea Ozaki, 1925. Moreover, recent molecular findings have reinforced the latter consideration [5].

According to Cribb [2], the subfamily level within the Opecoelidae is complex and unsatisfactory. He recognized at the time four subfamilies, namely the Opecoelinae Ozaki, 1925; Opecoelininae Gibson & Bray, 1984; Plagioporinae Manter, 1947 and Stenakrinae Yamaguti, 1970, which dominated the understanding of opecoelid taxa arrangement until the end of the twentieth century. However, phylogenetic studies [6,7,8] suggested that this subfamily classification was problematic and required significant revision. Thus, the subfamily classification of the Opecoelidae has vastly improved in recent years. Bray et al. [1] created a new opecoelid subfamily, the Helicometrinae Bray, Cribb, Littlewood & Waeschenbach, 2016 to accommodate the genera *Helicometra* Odhner, 1902; *Helicometrina* Linton, 1910; *Neohelicometra* Siddiqi & Cable, 1960 and *Proneohelicometra* Hassanine, 2006, species of which have unique egg- and uterus-related characters. Moreover, these authors demonstrated that the Opistholebetidae are deeply nested within the Opecoelidae and reduced the opistholebetids to a subfamily level [1]. Recently, Martin et al. [9] expanded the concept of the Opistholebetinae with the inclusion of the new genus *Magnaosimum* Martin, Crouch, Cutmore & Cribb, 2018, as well as other genera. This expanded concept is well supported by phylogenetic, morphologic and biological aspects [9]. Martin et al. [10] proposed the new subfamily Polypipapiliotrematinae Martin, Cutmore & Cribb, 2018 for the new genus *Polypipapiliotrema* Martin, Cutmore & Cribb, 2018, a parasite of butterflyfishes that includes the involvement of infected coral polyps in its life cycle. In addition, the subfamilies Bathycreadiinae Martin, Huston, Cutmore & Cribb, 2018 and Neopycnadeninae Karar, Blend, Mohamadain, Hassan, Khalifa & Dronen, 2020 were created to accommodate single genera, *Bathycreadium* Kabata, 1961 and *Neopycnadena* Karar, Blend, Mohamadain, Hassan, Khalifa & Dronen, 2020, respectively [11,12]. The subfamily Podocotylinae Dollfus, 1959 was resurrected and includes *Podocotyle* Dujardin, 1845 as the type genus as well as other genera [11]. Recently, Martin et al. [13] created the Pseudoplagioporinae Martin, Cutmore & Cribb, 2019 to include the plagioporines of the genus *Pseudoplagioporus* Yamaguti, 1938; *Fairfaxia* Cribb, 1989 and *Shimazuia* Cribb, 2005. The subfamily Hamacreadiinae Martin, Downie & Cribb, 2020 was created for the inclusion of species that use marine fishes as second intermediate hosts in their life cycles [14]. Recently, Sokolov et al. [15] elevated the Stenakridae Yamaguti, 1970 from subfamily to family rank to be included in the Opecoeloidea, and also proposed the new family Zdzitowieckitrematidae Sokolov, Shchenkov, Frolov & Gordeev, 2022, recognizing it in the Opecoeloidea. Moreover, these authors also proposed the new subfamily Scorpidotrematinae Sokolov, Shchenkov, Frolov & Gordeev, 2022 to accommodate the genera *Holsworthotrema* Martin, Huston, Cutmore & Cribb, 2018 and *Scorpidotrema* Aken’Ova & Cribb, 2003, previously considered stenakrines. Thus, according to the modern concept, which is based on phylogenetic and morphological data, the family Opecoelidae is currently divided into 12 subfamilies, namely the Bathycreadiinae; Hamacreadiinae; Helicometrinae; Neopycnadeninae; Opecoelinae; Opecoelininae; Opistholebetinae Fukui, 1929; Plagioporinae; Podocotylinae; Polypipapiliotrematinae; Pseudoplagioporinae and Scorpidotrematinae [10,11,12,13,14,15,16,17].

Combining multiple sources of information (morphological characters, molecular data, life cycle and the ultrastructural organization of male gametes) would allow for more robust phylogenetic inferences. In the case of the Opistholebetinae, the organization of sperm cells of representatives of this subfamily might provide additional information to clarify the systematic status and phylogenetic relationships within opecoelids, as demonstrated in other parasitic Platyhelminthes, especially monogeneans and cestodes [18,19,20,21,22,23,24,25,26,27]. Sperm ultrastructure has already been described for twelve opecoelid species distributed over five subfamilies. They are three Hamacreadiinae, namely *Allopodocotyle pedicellata* (Stossich, 1887), *Allopodocotyle tunisiensis* Derbel & Neifar, 2009 and *Podocotyloides magnatestis* Aleshkina & Gaevskaya, 1985 [28,29,30]; two Helicometrinae, namely *Helicometra epinepheli* Yamaguti, 1934 and *Helicometra fasciata* (Rudolphi, 1819) [31,32]; three Opecoelinae, namely *Labracetabulum gephyroberici* Reimer, 1987, *Opecoeloides furcatus* (Bremser in Rudolphi, 1819) and *Poracanthium furcatum* Dollphus, 1948 [33,34,35]; two Opistholebetinae, namely *Heterolebes maculosus* Ozaki, 1935 and *Macvicaria obovata* (Molin, 1859) [23,36] and two Plagioporinae, namely *Nicolla testiobliqua* (Wisniewski, 1933) and *Nicolla wisniewskii* (Slusarski, 1958) [37,38].

This study presents the first description of the sperm cell of the opistholebetine *Peracreadium characis* (Stossich, 1886), thus increasing the information on sperm ultrastructure in the Opecoelidae. Furthermore, some criteria potentially useful for phylogenetic analysis are discussed by comparing the obtained results with data from other Digenea, particularly other opecoelidean species.

## 2. Materials and Methods

### 2.1. Specimens

**Figure 1:**
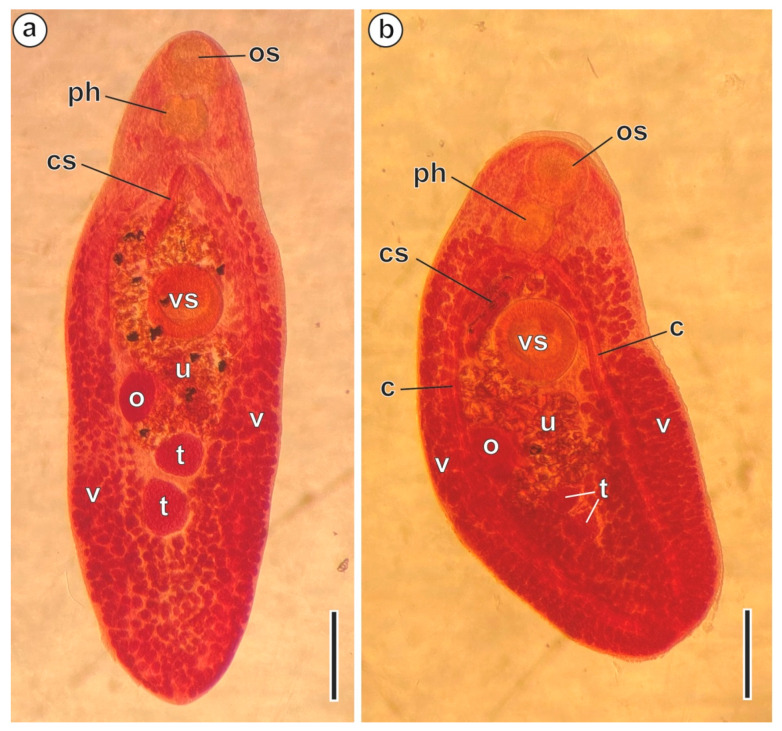
*Peracreadium characis* ex. *Diplodus puntazzo* from La Chebba (Tunisia) stained with Semichon’s acetic carmine. (**a**) Specimen no. 2018050708 (MNHN HEL1931). (**b**) Specimen no. 2018050607 (MNHN HEL1930). (c) caeca; (cs) cirrus-sac; (o) ovary; (os) oral sucker; (ph) pharynx; (t) testes; (u) uterus; (v) vitelline follicles; (vs) ventral sucker. Scale bars = 500 µm.

Fifty-two sheephead breams *Diplodus puntazzo* (Walbaum, 1792), captured in the Mediterranean Sea off La Chebba (34°14′ N, 11°06′ E) (Tunisia) in September 2016, March 2017 and May 2018, were scanned for helminths in their digestive system. Live adult digeneans were isolated from the intestinal tract, and some of them were stained with Semichon’s acetic carmine and mounted in Canada balsam (Figure 1a,b). The mounted specimens were then identified as *P. characis* in agreement with the specialized literature [2,39].

Two slides with three specimens of *P. characis* ex. *D. puntazzo* from La Chebba (Tunisia) were deposited in the Muséum National d’Histoire Naturelle (Paris, France) under the accession numbers MNHN HEL1930 and MNHN HEL1931.

### 2.2. Transmission Electron Microscopy

Living adult digeneans were rinsed immediately upon removal from the fish with a 0.9% NaCl solution. They were then fixed in 2.5% glutaraldehyde at 4 °C in a 0.1 M sodium cacodylate buffer (pH 7.4) for a period no shorter than 2 h. After rinsing in a 0.1 M sodium cacodylate buffer (pH 7.4), the digeneans were postfixed in 1% osmium tetroxide at 4 °C with 0.9% potassium ferricyanide in the same buffer for 1 h. After rinsing in Milli-Q water (Millipore Gradient A10, Millipore Co., Merck KGaA, Darmstadt, Germany), the dehydration process began using an ethanol series and propylene oxide. The specimens were finally embedded in Spurr’s epoxy resin and polymerized at 60 °C for 72 h. Ultrathin sections 60 nm thick were obtained using a Leica Reichert-Jung Ultracut E ultramicrotome (Leica Microsystems, Wetzlar, Germany), placed on copper grids and double-stained with uranyl acetate and lead citrate, as in Reynolds [40]. Stained grids were observed under a JEOL 1010 transmission electron microscope (JEOL Ltd., Tokyo, Japan), operated at an accelerating voltage of 80 kV, in the Centres Científics i Tecnològics de la Universitat de Barcelona (CCiTUB).

### 2.3. Cytochemistry

The Thiéry [41] technique was applied to allow for the cytochemical detection of glycogen. Ultrathin sections in gold grids were treated in periodic acid, thiocarbohydrazide and silver proteinate (PA-TCH-SP) in the following sequence: 30 min in 10% PA, rinsed in Milli-Q water, 24 h in TCH, rinsed in acetic solutions and Milli-Q water, 30 min in 1% SP in the dark and rinsed in Milli-Q water. Sections were examined with a JEOL 1010 transmission electron microscope (JEOL Ltd.) in the CCiTUB.

## 3. Results

**Figure 2:**
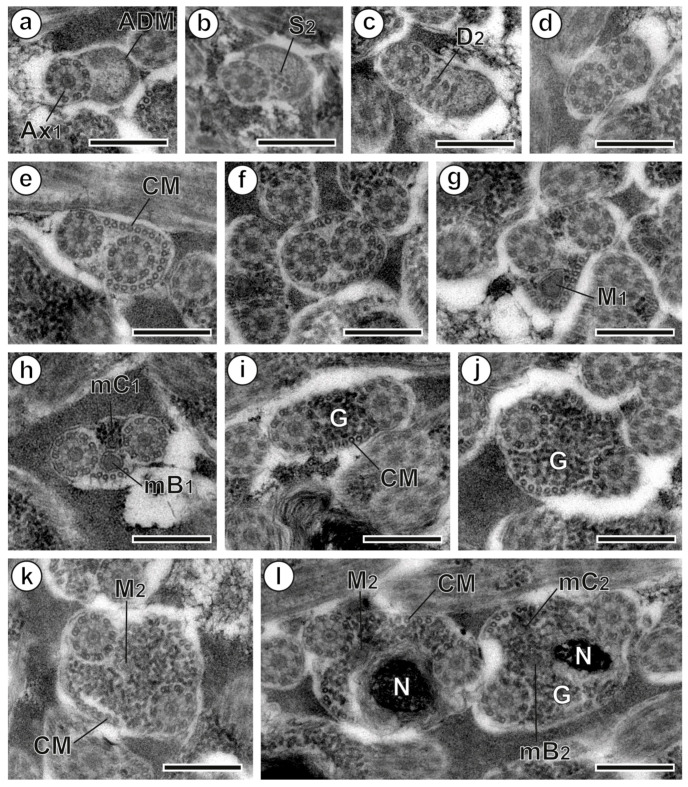
Spermatozoon of *Peracreadium characis*, regions I to III. (**a**) Cross-section of region I near the anterior spermatozoon extremity, showing the first axoneme (Ax_1_) and the anterior electron-dense material (ADM). (**b**–**d**) Correlative sections of region I illustrating the appearance of the second axoneme and the nucleus. (**e**,**f**) Sections of region I presenting the maximum number of cortical microtubules (CMs). (**g**,**h**) Sections of the posterior part of region I showing the first mitochondrion (M_1_). (**i**,**j**) Sections of the anterior part of region II. (**k**) Section of the posterior part of region II with the second mitochondrion (M_2_). (**l**) Sections of the anterior part of region III showing the simultaneous presence of the second mitochondrion and the nucleus (N). (D_2_) doublets of the second axoneme; (G) granules of glycogen; (mB_1_) bulge of the first mitochondrion; (mB_2_) bulge of the second mitochondrion; (mC_1_) cord of the first mitochondrion; (mC_2_) cord of the second mitochondrion; (S_2_) singlets of the second axoneme. Scale bars = 300 nm.

**Figure 3:**
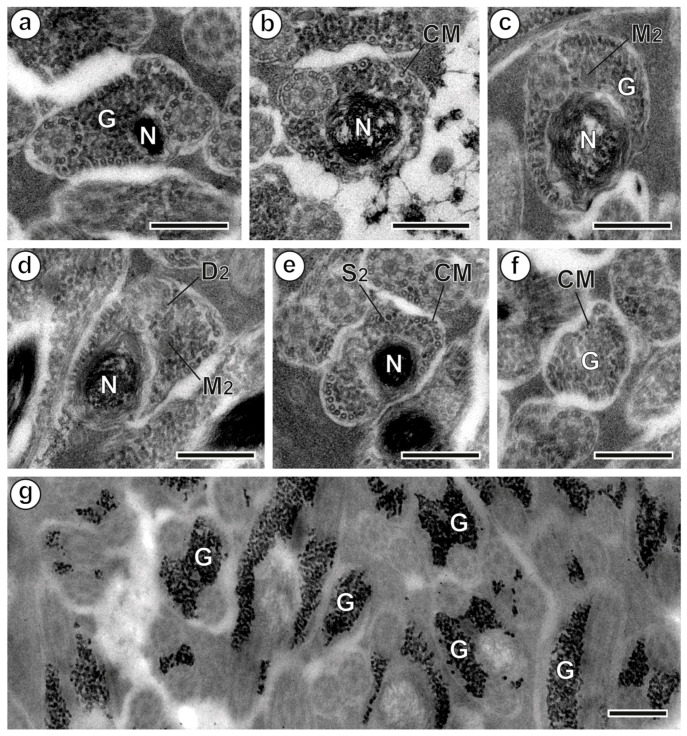
Spermatozoon of *Peracreadium characis*, region III. (**a**) Cross-section with the presence of both axonemes and nucleus (N). (**b**) Section showing the second axoneme and nucleus. (**c**) Section presenting the second axoneme, the second mitochondrion (M_2_) and the nucleus. (**d**,**e**) Sections showing the progressive disorganization of the second axoneme and the disappearance of the second mitochondrion. (**f**) Section near the posterior tip of the spermatozoon showing the sole presence of cortical microtubules (CM) and granules of glycogen (G). (**g**) Positive reaction of the Thiéry test for glycogen detection. The transition of sections toward the posterior spermatozoon extremity is a-b-e-f for posterior extremity type 1 and c-d-e-f for posterior extremity type 2. (D_2_) doublets of the second axoneme; (S_2_) singlets of the second axoneme. Scale bars = 300 nm.

The ultrathin sections of *P. characis* spermatozoa reveal three distinct regions (I, II and III) from their proximal to distal extremities, each with distinct ultrastructural characters (Figure 1, Figure 2 and Figure 3). The cell is filament-shaped, with structures found in many digeneans, including two axonemes, mitochondria, two bundles of parallel cortical microtubules, cytoplasm with glycogen granules and a nucleus.

### 3.1. Region I: Anterior Spermatozoon Extremity (Figure 2a–h and Figure 4I)

**Figure 4:**
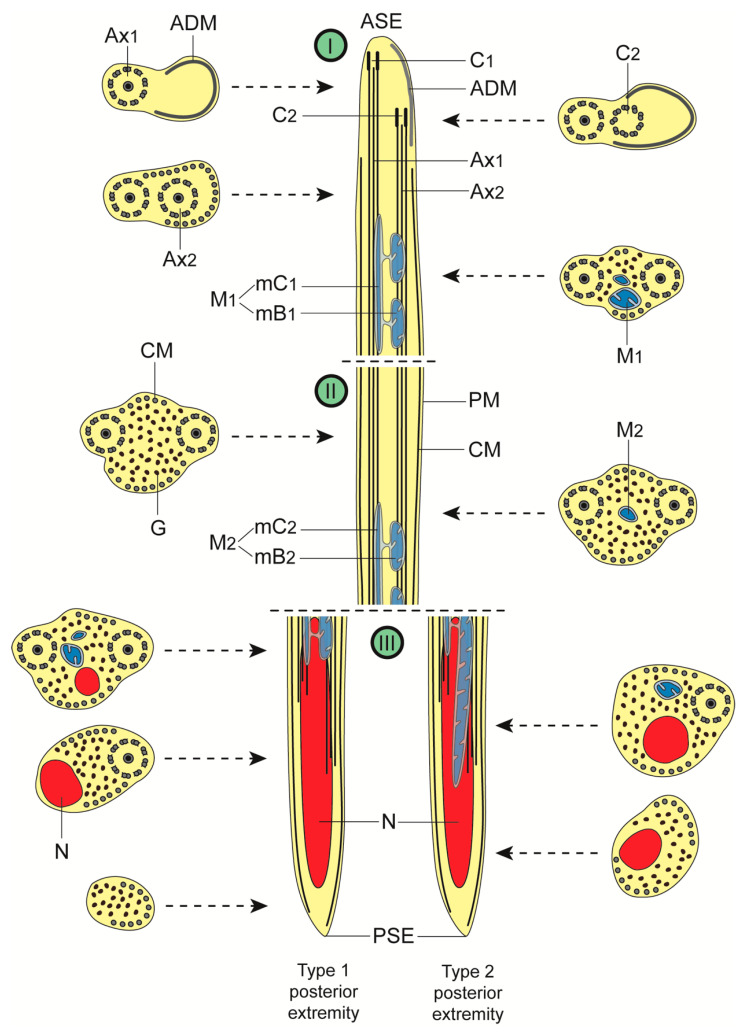
Schematic diagram illustrating the ultrastructural organization of the spermatozoon of *Peracreadium characis*. In order to make the diagram clearer, granules of glycogen are not shown in longitudinal sections. The variability observed in the transition of characters toward the posterior extremity of the spermatozoon is illustrated by two longitudinal sections of region III (type 1 and type 2). (ADM) anterior dense material; (ASE) anterior spermatozoon extremity; (Ax_1_) first axoneme; (Ax_2_) second axoneme; (C_1_) centriole of the first axoneme; (C_2_) centriole of the second axoneme; (CM) cortical microtubules; (G) granules of glycogen; (**I**) anterior spermatozoon region; (**II**) middle spermatozoon region; (**III**) posterior spermatozoon region; (M_1_) first mitochondrion; (M_2_) second mitochondrion; (mB_1_) bulge of the first mitochondrion; (mB_2_) bulge of the second mitochondrion; (mC_1_) cord of the first mitochondrion; (mC_2_) cord of the second mitochondrion; (N) nucleus; (PM) plasma membrane; (PSE) posterior spermatozoon extremity.

The region near the anterior tip of the sperm cell displays one 9+‘1’ trepaxonematan axoneme as well as an electron-dense material, which disappears when the second axoneme’s singlets and doublets appear (Figure 2a–c and Figure 4I). A layer of submembranous cortical microtubules appears posteriorly to the formation of the second axoneme (Figure 2d,e), and initially they surround the second axoneme (Figure 2e). The greatest number of cortical microtubules (around 21 elements) occurs at this level before their distribution into two bundles with a lower number of microtubules [15, 5 dorsal + 10 ventral (Figure 2f); 18, 4 + 14 (Figure 2g); 7, 2 + 5 (Figure 2h)]. The first mitochondrion is observed in the distal portion of region I (Figure 2g,h). This mitochondrion is moniliform, in the shape of a cord with linked mitochondrial bulges (Figure 2g,h and Figure 4I). Additionally, this portion of the cell exhibits glycogen granules, as evidenced by the Thiéry test (Figure 3g). The disappearance of the first mitochondrion marks the transition to region II.

### 3.2. Region II: Middle Spermatozoon Region (Figure 2i–k and Figure 4II)

The middle region or region II has two axonemes, parallel cortical microtubules arranged into two bundles and a large amount of glycogen granules in its anterior part (Figure 2i,j and Figure 4II). Another moniliform mitochondrion can be observed in the posterior portion of region II (Figure 2k and Figure 4II). It is noteworthy that the number of cortical microtubules increases from 8 (7 + 5) (Figure 2i) to 15 (7 + 8) (Figure 2k).

### 3.3. Region III: Posterior Spermatozoon Extremity (Figure 2l, Figure 3a–f and Figure 4III)

The proximal part of region III starts with the appearance of the nucleus, which is accompanied by the simultaneous presence of the second mitochondrion and the two axonemes, glycogen granules and cortical microtubules (Figure 2l, Figure 3a and Figure 4III). The character sequence transition toward the posterior tip of the spermatozoon varies among sperm cells. In fact, the second mitochondrion disappears at different levels, either before or after the first axoneme becomes disorganized. As represented in Figure 4III, one type of posterior extremity is characterized by the following sequence of character disappearance: second mitochondrion, first axoneme, second axoneme, nucleus and cortical microtubules (Figure 3a,b,e,f and Figure 4III). A second type exhibits the following sequence of character disappearance: first axoneme, second axoneme, second mitochondrion, nucleus and cortical microtubules (Figure 3c–f and Figure 4III). The posterior tip of the spermatozoon has only cortical microtubules and granules of glycogen.

## 4. Discussion

The mature spermatozoa of *P. characis* are filiform cells tapered at both ends, which share the common ultrastructural features described in the majority of digeneans: two axonemes, a nucleus, mitochondria and parallel cortical microtubules. However, compared to most opecoelidean species studied, the mature spermatozoon of *P. characis* presents some unusual features that are briefly discussed below.

### 4.1. Anterior Extremity: Anterior Dense Material

In *P. characis,* the spermatozoon’s anterior extremity displays the first axoneme with a continuous and submembranous layer of an electron-dense material located on the opposite side of the axoneme, which disappears when the two axonemes are completely formed and the cortical microtubules appear. This structural element has been described in all opecoelids studied up to now, except in the opistholebetine *Heterolebes maculosus*, the opecoeline *Opecoeloides furcatus* and the plagioporines *Nicolla testiobliqua* and *N. wisniewskii* [23,34,37,38].

Apart from the Opecoelidae, the presence of anterior dense material has also been reported in numerous other species of other families, particularly in the Aephnidiogenidae Yamaguti, 1934 [42,43,44]; the Emprostiotrematidae Cianferoni & Ceccolini, 2021 [45]; the Gyliauchenidae Fukui, 1929 [24,46]; the Lepocreadiidae Odhner, 1905 [47,48,49,50,51] and the Cryptogonimidae Ward, 1917 [52]. It is important to note the morphological variability of this structural element. It appears as a discontinuous submembranous layer of electron-dense material on the opposite side of the first axoneme beneath the plasma membrane in several digenean species. However, in mature spermatozoa of the lepocreadiids *Bianium arabicum* Sey, 1996 and *B. plicitum* (Linton, 1928), this layer appears as a mass of electron-dense material located within a cytoplasmic expansion [50].

### 4.2. Axonemes

The axoneme is a significant source of characters for elucidating platyhelminth phylogeny. The shape and length of the axoneme have been the subject of numerous discussions as spermatological characters in Digenea phylogeny. In terms of their morphology, the spermatozoon of *P. characis* possesses two axonemes exhibiting the 9+‘1’ trepaxonematan pattern, which is composed of nine peripheral doublets with dynein arms and a highly structured central element [53]. This type of axoneme has been observed in the majority of Digenea sperm cells, with the exception of species of *Schistosoma* Weinland, 1858 (Schistosomatidae Stiles & Hassall, 1898) and *Didymozoon* Taschenberg, 1879 (Didymozoidae Monticelli, 1888): the former presents the 9+‘1’ special pattern (one diffuse central element and nine peripheral doublets with no dynein arms) [54], while the latter presents the 9+0 axoneme pattern (nine doublets with neither outer dynein arms nor central structure) [55].

The axonemes of the male gamete of *P. characis* present different lengths, as in all opecoelid spermatozoa studied until now. In this species, the disorganization of the first axoneme occurs at the nuclear region of the spermatozoon, similarly to all Opecoelidae species except for *Labracetabulum gephyroberici* and *O. furcatus* [33,34], in which the first axoneme disorganizes and disappears at the very anterior part of the nuclear region. Within the Digenea, only the lecithasterid *Aponurus laguncula* Looss, 1907 and the haplosplanchnid *Haplosplanchnus caudatus* (Srivastava, 1937) [56,57] show two axonemes with the same length presenting centrioles at the same level in the anterior spermatozoon extremity, and both axonemes also disorganize at the same level in the posterior part of the nuclear region.

### 4.3. External Ornamentation

An external ornamentation of plasma membrane has been reported in the anterior areas of male gametes in many species of the digeneans. This ornamentation does not usually cover the entire perimeter of the cell [26]. The location of the external ornamentation along the spermatozoon varies among species, leading to three groups of digeneans: a group with ornamentation in the apical spermatozoan region; a group with a more posterior external ornamentation, most commonly in the mitochondrial region and another group that includes species presenting spermatozoa without external ornamentation [24]. The spermatozoon of *P. characis* such as *Helicometra fasciata* lacks external ornamentation, following Quilichini et al.’s [24] type 3 spermatozoon, in contrast with the remaining Opecoelidae studied to date, which exhibit external ornamentation in the mitochondrial region corresponding to Quilichini et al.’s [24] type 2.

### 4.4. Cortical Microtubules

Different aspects of cortical microtubules are considered crucial in platyhelminth phylogeny inference, including whether they are present or not, how they are aligned, the region where their maximum number is located, the quantity of bundles and if they are associated or not with the external ornamentation of the plasma membrane [26].

Cortical microtubules form a tubular structure totally or partially underlying the plasma membrane in most digenean spermatozoa, except for *Didymosulcus wedli* (Ariola, 1902) (studied as *Didymocystis wedli* Ariola, 1902) and *Didymozoon* sp. sperm cells, which are devoid of cortical microtubules [55,58].

The parallel alignment of cortical microtubules has also been found in the sperm cells of most groups of parasitic flatworms, such as the Monogenea van Beneden, 1858 (except in certain monopisthocotyleans) [27,59,60] and the Eucestoda Southwell, 1930, except for the Cyclophyllidea van Beneden in Braun, 1900 and Tetrabothriidea Baer, 1954, which have twisted cortical microtubules [25].

Among the majority of digeneans, the cortical microtubules are typically arranged into two bundles in the sperm cell. This was also observed in *P. characis,* similarly to other opecoelidean species studied to date. However, some hemiuroidean species (such as the lecithasterids, hemiurids and sclerodistomids) exhibit only one bundle of cortical microtubules on the ventral or mitochondrial side of the sperm cell [56,61,62,63].

Another aspect of interest in the phylogenetic study of digeneans is the location of the maximum number of cortical microtubules in sperm. Based on the position of these microtubules, two groups of digeneans can be distinguished [26,37]: a group where the highest number of cortical microtubules is located in the anterior region of the spermatozoon and a group where the highest number of cortical microtubules is situated at an intermediate region of the sperm cell (respectively classified as type 1 and 2 according to Quilichini et al. [37]).

In *P. characis,* the maximum number of cortical microtubules is located in the anterior part of the spermatozoon, representing a type 1 spermatozoon according to Quilichini et al. [37]. This type is present in *Allopodocotyle pedicellata* and *A. tunisiensis* [28,29]. The remaining Opecoelidae species, except for *Macvicaria obovata*, exhibit a type 2 sperm cell, with the maximum number of cortical microtubules located in the middle part of the spermatozoon (see Table 1). In *M. obovata,* the maximum number of cortical microtubules is located in the nuclear region of the spermatozoon after the disappearance of the first axonemes [36].

### 4.5. Spine-Like Bodies

These triangular, electron-dense prominences containing a submembranous vesicle were first described in the opecoelid species *O. furcatus* by Miquel et al. [34]. The variability of their presence, absence, number, size and location in ornamented and/or non-ornamented areas has been widely discussed [26]. They are frequently observed in mature sperm cells of digenean species, including those in the Opecoelidae family (Table 1). However, spine-like bodies are absent from male gametes of *H. fasciata* [32] and *P. characis* (present study).

### 4.6. Mitochondrion

Mitochondria are a crucial component in digenean spermatozoa and are used to determine phylogenetic relationships. Their presence is considered a plesiomorphic character, while their absence is seen as a synapomorphy for the Eucestoda [18]. The formation of mitochondria in spermatozoa occurs during spermiogenesis, when numerous mitochondria accompany the nucleus’ migration along the median cytoplasmic process [64]. The number, shape and location of mitochondria can be used to make phylogenetic inferences. The number of mitochondria in mature spermatozoa can range from one to three [26]. In the case of *P. characis*, male gametes have two mitochondria, in agreement with most studies on Opecoelidae spermatozoa. To date, the exception is the male gamete of *O. furcatus* and *H. fasciata* exhibiting only one mitochondrion [32,34].

The shape of mitochondria is a feature that varies among members of the Opecoelidae family. Different morphological types have been described, including tubular, moniliform and U-shaped posterior extremities. Bâ et al. [41] were the first to describe a moniliform mitochondrion in *Holorchis micracanthum* (Stossich, 1889), which appears as a series of mitochondrial bulges joined by a cord. This shape has also been reported in the spermatozoon of *A. pedicellata* and *M. obovata* [28,36]. Finally, a U-shaped posterior extremity of mitochondrion was described in *A. tunisiensis* by Kacem et al. [29].

It is important to note that moniliform mitochondria have been reported in spermatozoa of species other than opecoelids, including the sclerodistomoidid *Sclerodistomoides pacificus* Kamegai, 1971 [65], aephnidiogenids of the genus *Holorchis* Stossich, 1901 [42,44], the lepocreadiids *Opechona bacillaris* (Molin, 1859) and *Prodistomum polonii* (Molin, 1859) [49,51], the cryptogonimids *Aphallus tubarium* (Rudolphi, 1819) and *Timoniella imbutiformis* (Molin, 1859) [66,67] or the plagiorchiid *Enodiotrema reductum* Looss, 1901 [68]. Nevertheless, the present study in *P. characis* constitutes the first description of two moniliform mitochondria in a sperm cell.

The placement of mitochondria is considered an interesting aspect in most digeneans. In *O. furcatus* and *H. fasciata* [32,34], a single mitochondrion overlaps the anterior portion of the nucleus in the middle region of the cell. It always disappears before the posterior end of the second axoneme. In the remaining Opecoelidae species, two mitochondria are present: the first is located in the ornamented area of the spermatozoon and the second in the nuclear region. The level of their disappearance varies among the studied Opecoelidae species.

*Peracreadium characis* exhibits variability in the level of disappearance of the second mitochondrion, which can occur either before or after the posterior end of the second axoneme. This variability in the disappearance level of the second mitochondrion has also been described in *A. tunisiensis* [29], where it can occur both before and after the disorganization of the first axoneme. In *L. gephyroberici* and *P. magnatestis* [30,33], the second mitochondrion disappears after the posterior end of the second axoneme. In *A. tunisiensis* and *H. epinepheli* [29,31], it disappears at the same level as the second axoneme. In the other studied Opecoelidae, the second mitochondrion disappears before the posterior end of the second axoneme [23,28,35,36,37,38].

### 4.7. Posterior Region of the Spermatozoon

The distal region of the sperm cell presents a highly variable morphology, and is considered to be a significant feature for establishing sperm models in digenean parasites.

In fact, Quilichini et al. [23] proposed three types of posterior spermatozoon extremities defined by the transition sequence of the second axoneme, cortical microtubules and nucleus. The Opecoelidean or type 1 sperm extremity presents the sequence ‘axoneme, nucleus and cortical microtubules’, whereas the Fasciolidean or type 2 sperm extremity presents the sequence ‘cortical microtubules, axoneme and nucleus’. Finally, the Cryptogonimidean or type 3 extremity presents the sequence ‘cortical microtubules, nucleus and axoneme’.

In *P. characis*, variability in the level of disappearance of the second mitochondrion can lead to variations in the sequence of sperm components toward the posterior extremity of the spermatozoon. This disappearance can occur either before or after the posterior end of the second axoneme. In fact, due to the non-conformity of posterior spermatozoon extremities of several species of digeneans to any of models proposed in Quilichini et al. [23], Bakhoum et al. [26] proposed other types of posterior spermatozoon extremities defined only by the last spermatozoon character instead of the sequence of characters in the extremities. Thus, according to Bakhoum et al. [26], the posterior extremity of the male gamete of *P. characis* belongs to type characterized by the presence of only cortical microtubules in the posterior extremity of the spermatozoon. This is a feature observed in the mature sperm of all opecoelids studied so far, except for *P. magnatestis* [30], which exhibits the nucleus.

## 5. Concluding Remarks: Contribution of Spermatological Characteristics to Opecoelidean Phylogenetic Inferences

Concerning the relationship between Opecoelidae and Brachycladioidea, differences in spermatozoa ultrastructural organization were reported between Opecoelidae and Acanthocolpidae (*Stephanostomum murielae* Bray & Justine, 2011 and *Stephanostomoides tenuis* (Manter, 1963) [69]), which are members of the Brachycladioidea. Therefore, the analysis of ultrastructural data of spermatozoa do not support a close relationship between the two groups.

Regarding the structure and organization of sperm cells in opecoelids, it has been found that they are quite similar. However, there are several discrepancies when analyzing the existing results in the different subfamilies. With regard to external ornamentation, all species follow pattern 2 as defined by Quilichini et al. [24], with the exception of *P. characis* (present study) and *H. fasciata* [32]. As for the posterior extremity, all studied species exhibit the opecoelidean type established by Quilichini et al. [23], with cortical microtubules as the terminal character, except for *P. magnatestis* [30], which is defined by the presence of the nucleus in the posterior tip of the male gamete. Despite the presence of anterior dense material in several opecoelid species, this character is absent in *H. maculosus*, *N. testiobliqua*, *N. wisniewskii* and *O. furcatus* [23,34,37,38].

Considering the digenean spermatozoa models proposed in Bakhoum et al. [26], model III describes the typical Opecoelidae sperm cell type. It is noteworthy that this pattern cannot be attributed to *Allopodocotyle* species (Hamacreadiinae) [28,29] because the largest number of cortical microtubules is located in an intermediate area of the sperm cell. Therefore, the studied *Allopodocotyle* species agree with the type IV model of digenean sperm cells. However, the other hamacreadiine studied to date (*P. magnatestis*) exhibits the type III spermatozoon [30]. In the subfamily Opistholebetinae, the studied species also show variability in the spermatozoon model. Thus, the sperm cell of *M. obovata* follows the type III whereas that of *P. characis* follows the type IV. The third studied opistholebetine, *H. maculosus*, has a doubtful or questionable location of the maximum number of cortical microtubules [23]. The remaining analyzed subfamilies, namely the Helicometrinae, Opecoelinae and Plagioporinae, are homogeneous in relation to the pattern of the sperm cell. The similarity in the ultrastructure of spermatozoa between *P. characis* (present study) and *Allopodocotyle* species [28,29] suggests a close relationship between the Opistholebetinae and Hamacreadiinae subfamilies, and this finding is supported by genetic studies conducted by Sokolov et al. [15]. However, using more extensive data, the phylogenetic analyses performed by Martin et al. [13] are more robust and do not show a close relationship between these subfamilies.

## Figures and Tables

**Table 1:** Ultrastructural characteristics of the spermatozoon in the Opecoelidae.

Subfamilies and Species	Principal Characters								Secondary Characters			TSpz	References
	TAx	LE	EO	EO+CM	LEO	BCM	LMCM	M	ADM	SB	PSC		
**Hamacreadiinae**													
*Allopodocotyle pedicellata*	9+‘1’	−	+	+	PostA	2	AntS	2	+	+	CM	IV	[28]
*Allopodocotyle tunisiensis*	9+‘1’	−	+	+	PostA	2	AntS	2	+	+	CM	IV	[29]
*Podocotyloides magnatestis*	9+‘1’	−	+	+	PostA	2	MedS	2	+	+	N	III	[30]
**Helicometrinae**													
*Helicometra epinepheli*	9+‘1’	−	+	+	PostA	2	MedS	2	+	+	CM	III	[31]
*Helicometra fasciata*	9+‘1’	−	−	NA	NA	2	MedS	1	+	−	CM	III	[32]
**Opecoelinae**													
*Labracetabulum gephyroberici*	9+‘1’	−	+	+	PostA	2	MedS	2	+	+	CM	III	[33]
*Opecoeloides furcatus*	9+‘1’	−	+	+	PostA	2	MedS	1	−	+	CM	III	[34]
*Poracanthium furcatum*	9+‘1’	−	+	+	PostA	2	MedS	2	+	+	CM	III	[35]
**Opistholebetinae**													
*Heterolebes maculosus*	9+‘1’	−	+	+	PostA	2	MedS?	2	−	+	CM	III–IV?	[23]
*Macvicaria obovata*	9+‘1’	−	+	+	PostA	2	PostS	2	+	+	CM	III	[36]
*Peracreadium characis*	9+‘1’	−	−	NA	NA	2	AntS	2	+	−	CM	IV	Present study
**Plagioporinae**													
*Nicolla testiobliqua*	9+‘1’	−	+	+	PostA	2	MedS	2	−	+	CM	III	[37]
*Nicolla wisniewskii*	9+‘1’	−	+	+	PostA	2	MedS	2	−	+	CM	III	[38]

(ADM) anterior electron-dense material; (AntS) anterior region of the spermatozoon; (Ax) axoneme; (BCM) number of bundles of cortical microtubules; (CM) cortical microtubules; (EO) external ornamentation of the plasma membrane; (EO+CM) external ornamentation–cortical microtubule association; (LE) lateral expansion; (LEO) location of external ornamentation; (LMCM) location of maximum number of cortical microtubules; (M) number of mitochondria; (MedS) median region of the spermatozoon; (N) nucleus; (NA) not applicable; (PostA) posterior part of anterior region; (PostS) posterior region of the spermatozoon; (PSC) posterior spermatozoon character; (SB) spine-like bodies; (TAx) type of axoneme; (TSpz) type of spermatozoon according to Bakhoum et al. [26]; (+) presence of considered character; (−) absence of considered character; (?) doubtful or unknown data.

## Data Availability

The data presented in this study are available upon request from the corresponding author. The data are not publicly available due to internal laboratory policy.
